# Anisotropic Phase Transformation in B2 Crystalline CuZr Alloy

**DOI:** 10.1186/s11671-019-3116-6

**Published:** 2019-08-16

**Authors:** Shayuan Weng, Tao Fu, Xianghe Peng, Xiang Chen

**Affiliations:** 1 Department of Engineering Mechanics, Chongqing University, Chongqing, 400044 China; 2 State Key Laboratory of Coal Mine Disaster Dynamics and Control, Chongqing University, Chongqing, 400044 China; 3 Advanced Manufacturing Engineering, Chongqing University of Posts and Telecommunications, Chongqing, 400065 China

**Keywords:** B2 phase CuZr, Phase transformation, Molecular dynamics, Uniaxial loading, Anisotropic

## Abstract

B2 phase copper-zirconium (CuZr) particles are often used as an enhancement agent to improve the toughness of metallic glass; however, the orientation dependence of its phase transformation behaviors under loading remains unclear. In this work, molecular dynamics simulation of uniaxial tension and compression of B2 phase CuZr along different crystallographic orientation are performed to investigate the orientation-related mechanical response and phase transformation mechanisms. It was found that the mechanical behavior of CuZr exhibits obvious tension/compression asymmetry, but their failure mode is mainly local amorphization. Three different phase transformation behaviors, B2→FCC, B2→BCT, and B2→HCP, were observed in tension and compression along [001], and tension along [110], respectively. The transformations are realized by lattice rotation (~ 5°), uniform deformation and separation between Cu and Zr atomic layers, respectively. Before failure by local amorphization, phase transformation region can be recovered after unloading, showing the superelasticity.

## Introduction

Bulk metallic glasses (BMGs) have attracted considerable attention due to their excellent mechanical and physical properties, such as high strength, elasticity, high hardness combined with excellent corrosion resistance, etc. [1–4]. Nevertheless, they usually fail by catastrophic brittle fracture through localized shear bands [5, 6]. This shortcoming has been overcome to a certain extent in some CuZr-based bulk metallic glass composites (BMGCs) enhanced by ductile B2 phase CuZr particles [3, 7–11]. Moreover, some B2 crystalline CuZr precipitations would be inherently formed through the crystallization in CuZr glass under loading, and then undergo twinning and dislocation gliding, inducing the change in the mechanical properties of BMGs, as was found in the experiment [12]. To toughen BMGs with B2 phase CuZr precipitations and design high-performance BMGs, the deformation behaviors of B2 phase CuZr should be clarified firstly.

B2 phase CuZr is a kind of shape memory alloy that has the ability to recover its original shape under specific thermo-mechanical conditions [13, 14], which is different from the traditional crystalline materials that take dislocation glide or twinning as the main deformation mechanism [15–17]. The first-principle calculation based on functional density theory can be used to study the adsorption process [18–20] and the interfacial property [21, 22], but cannot be applied to study the dynamic evolution of the phase transition behaviors due to the limitation of calculation scale. Molecular dynamics (MD) simulation is an effective method to study the mechanical properties and deformation behaviors of materials [23–31]. Sutrakar and Mahapatra investigated the effects of cross-sectional dimensions and temperature on the phase transformation in Cu-Zr nanowire, as well as the tension-compression asymmetry by MD simulation [32–34], and obtained some valuable results. For instance, the initial B2 phase is transformed to a body-centered-tetragonal (BCT) phase by the nucleation and propagation of a {100} twinning plane. Amigo et al. used two kinds of potentials [35, 36] in their MD simulations to investigate the phase transformation behaviors, and found that one yields the martensitic transformation from B2 to BCT structure, while the other does not [13].

It is known that the anisotropy of crystals plays an important role in the deformation of materials. Different deformation mechanisms may play dominant roles during deformation when the load is applied along different crystal orientation [37]. For instance, perfect dislocation glide and twinning are the main deformation mechanisms for the nanoindentation on (001) and (111) surfaces of vanadium nitride (VN) with a cylindrical indenter [38, 39], respectively, showing obvious anisotropic plasticity. For BCC iron nanowire, the phase transformation shows an intricate dependence on the crystallographic orientation, along which the load is applied, <001> oriented wire exhibits a BCC→FCC transformation, but <011> and <111> oriented wire follows a BCC→HCP transformation [40]. The atomic distribution array in B2 structure is analogous to that in a BCC structure, but there are two kinds of elements in B2 structure. The crystal orientation of the reinforcing particles in BMGs is usually scattered, thus loading direction should have different influences on the toughening effect of different particle. Thus, it is necessary to study the deformation behavior of the enhancement particles with loading along different orientations.

Uniaxial tension and compression, as two basic loading modes usually used to evaluate the fundamental mechanical properties of materials. In this work, a series of MD simulations of uniaxial tension and compression tests of B2 crystalline CuZr along [001], [110], and [111] orientations are conducted to explore the dependence of phase transformation on loading orientation and the tension and compression (T/C) asymmetry.

## Methods

The well-known embedded-atom method (EAM) [41] is selected to describe the interatomic force of Cu-Zr system. The EAM potential has been widely used to investigate the mechanical behavior of metals and their alloys [42–46]. Based on the frame of EAM, Mendelev and his colleagues have identified and optimized the potential parameters three times in 2007 [35], 2009 [36], and 2016 [47]. In this work, the parameters in the latest version of the interatomic potential for Cu-Zr [47] developed in 2016 are used. These parameters can yield more realistic stable and unstable stacking fault energy compared with that developed in 2009 [36], and can better describe the properties of crystalline CuZr.

**Fig. 1:**
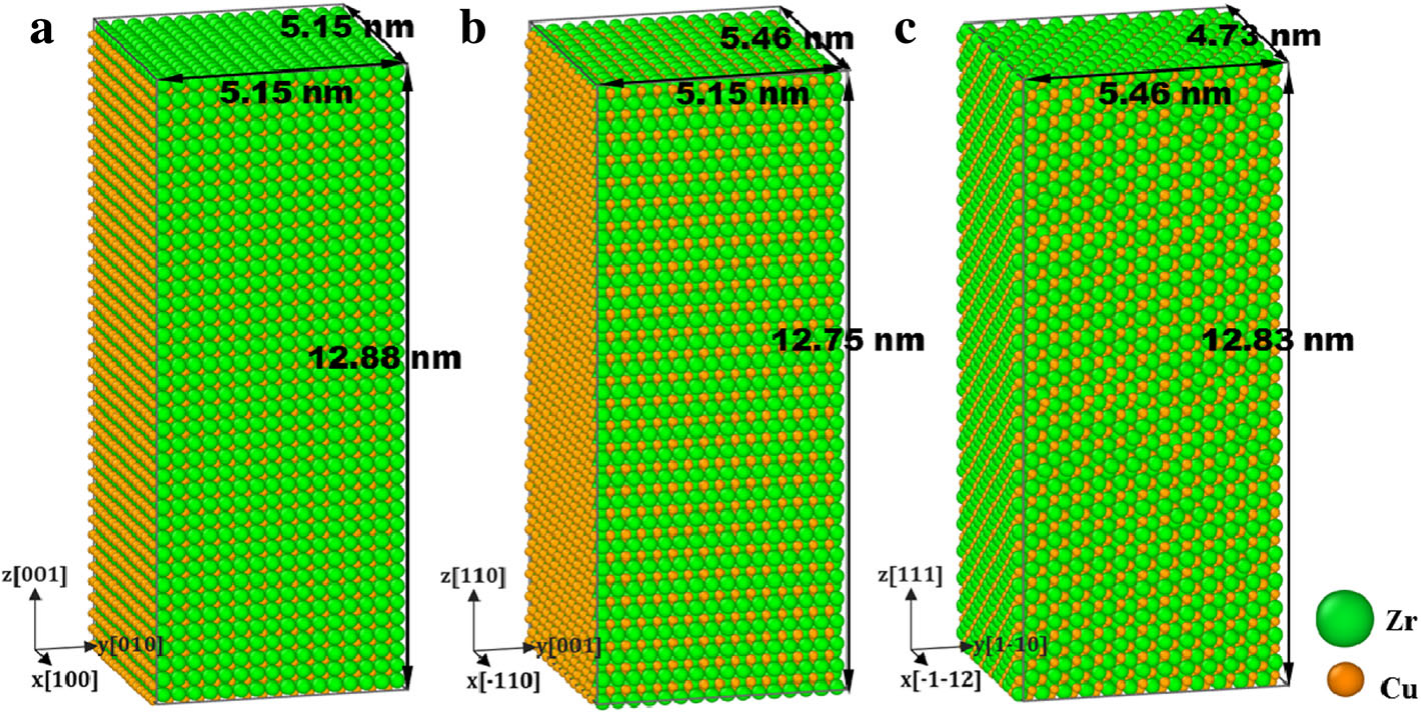
Samples with axial z along **a** [001], **b** [110], and **c** [111], colored with atomic type

Three samples with the loading axial *z* along the [001], [110], and [111] are prepared, respectively, as shown in Fig. 1. Before loading, a conjugate gradient (CG) algorithm is used to minimize the energy of the system to reach an optimized stable configuration. Mechanical tests are simulated at room temperature of 300 K. Then the system is relaxed with the isothermal-isobaric NPT ensemble at *T* = 300 K for 20 ps to reach an equilibration state with pressure-free condition. It is found that the strain rate effect of nano-polycrystalline material becomes insignificant as the strain rate varies in the range between 5 × 10^8^ and 1 ×10^9^ s^−1^, therefore the strain rate is assigned as 1 × 10^9^ s^−1^ by considering comprehensively both the accuracy and computation efficiency [48, 49]. Hence, each sample is stretched/compressed in the *z*-direction at a strain rate of 10^9^ s^−1^ during loading; meanwhile, the NPT ensemble with Nose/Hoover barostat is employed [50] to keep pressure free in *x*- and *y*-directions. In the relaxation and the loading stage, periodic boundary conditions are applied in *x*-, *y*-, and *z*-directions.

Common neighbor analysis (CNA) [51], an algorithm to characterize the local structural environment, is usually used as an effective method to classify the atoms in a crystalline system. The second nearest neighbor distance in a BCC structure is only 15% larger than the nearest one; therefore, CNA method would lose some reliability in the presence of strong thermal fluctuations and strain. To overcome this short, the polyhedral template matching (PTM) method was proposed to classify the local structural environment of particles and identify the local crystalline structure of simple condensed phases (FCC, BCC, HCP, etc.) [52]. Compared with CNA [51], the PTM method promises greater reliability in the presence of strong thermal fluctuations and strain [52]. B2 and BCC structures have an analogous atomic arrangement; therefore, the identified BCC structure by this method is actually the B2 structure. After the local microstructure analysis for the data obtained by MD simulation with PTM, the atoms are colored according to the following rules: blue for BCC (B2) structure, green for FCC structure, red for stacking faults or HCP structure, purple for simple cubic (SC), and white for grain boundaries or dislocation cores. It should be noted that single layer, double layer, and continuous multilayer red atoms are represented as twin boundary, stacking fault, and HCP structure, respectively. Local regions containing red, green, blue, and white atoms are amorphous.

As a supplementary microstructure analysis method, the centro-symmetry parameter (CSP) is used to describe the local disorder [53]. For each atom, the CSP is calculated with the flowing formula:

1 $$ \mathrm{CSP}\kern0.5em =\kern0.5em \sum \limits_{i=1}^{N/2}{\left|{\mathbf{R}}_i+{\mathbf{R}}_{i+N/2}\right|}^2, $$

where *N =* 12 or 8 is the number of the first nearest-neighbors of a central atom in FCC or B2 structure, and **R**_*i*_ and **R**_*i+N/2*_ are the vectors from the central atom to a particular pair of the nearest neighbors. The CSP is zero for an atom whose nearest-neighbors are at their perfect lattice sites. If there is a defect such as a vacancy or dislocation in the vicinity of an atom, the CSP of the atom will become much larger than that caused by local atomic vibration. The open software Ovito developed by Stukowski [54] is utilized to display atomic configurations.

## Results and Discussions

### Stress-Strain Curves

**Fig. 2:**
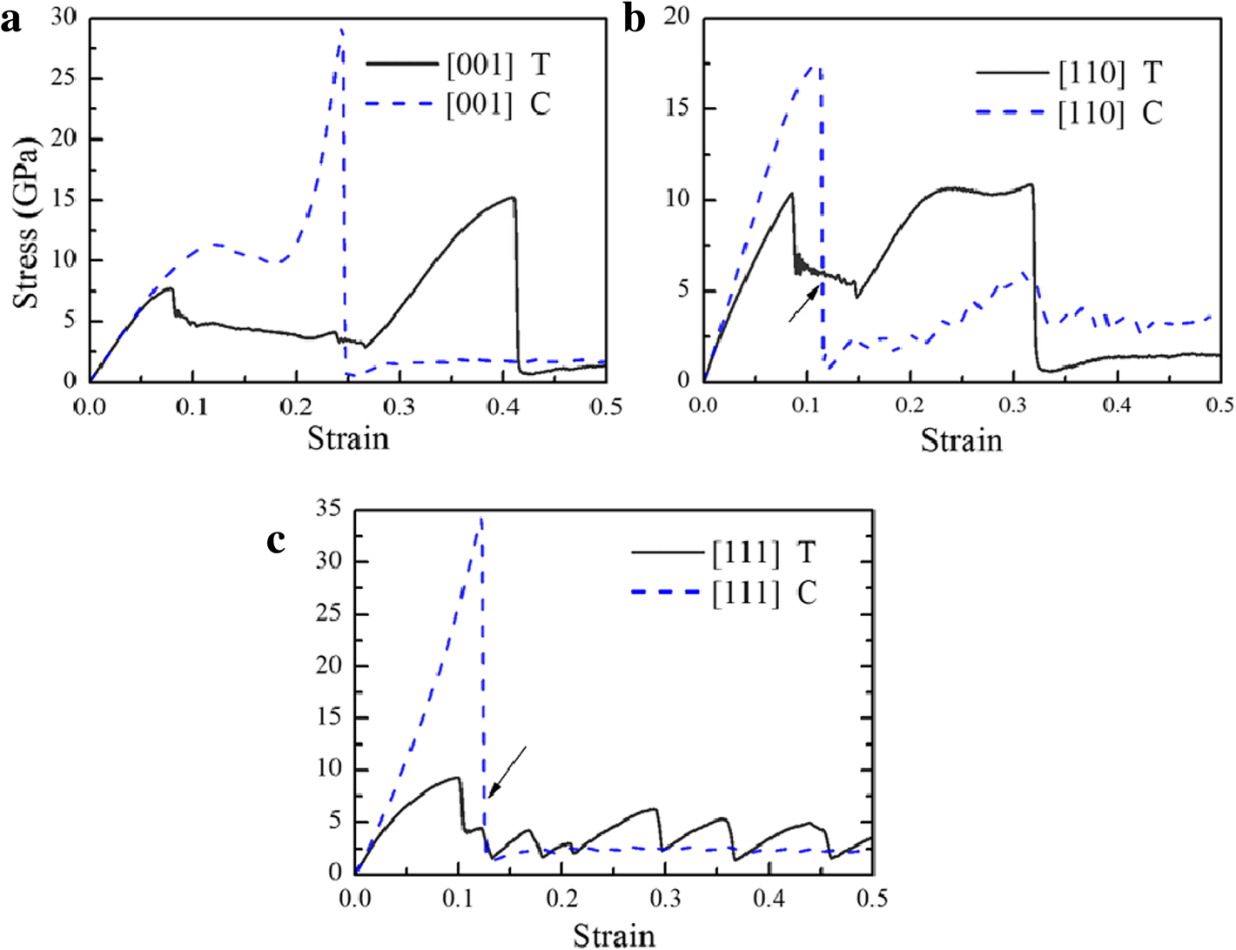
*σ*-*ε* curves of samples under tension (T) and compression (C). **a** [001], **b** [110] and **c** [111]

Figure 2 shows the stress-strain (*σ*-*ε*) curves for B2 phase CuZr subjected to uniaxial tension and compression along [001], [110], and [111]. It can be noted that the stress is larger than that by experiment [55], because (1) the time scale used in MD simulation differs from that used in experiment, resulting in a much larger indentation speed than that in experiment; and (2) the defects including point defect, dislocations, and grain boundaries etc. are not considered in the simulations. At the initial stage, these curves develop linearly and then show different trends. After the first peak, these curves can be divided into three groups. In group I, the curves drop rapidly to low levels of stress, such as compression along [110] and [111]. In group II, the stresses fall to a platform and fluctuate with the increase of strain after the first peak, such as tension along [001], [110] and compression along [001]. The curves then climb to their second peaks before final sharp drop. In group III, the curve drops rapidly to low levels of stress, and then fluctuates in a zigzag pattern, such as tension along [111]. Before the first peak, samples stay in the B2 structure and no obvious dislocation glide and twinning can be observed, which can be regarded as the elastic deformation. In the linear elastic stage, the Young’s modulus *E* can be obtained by fitting the slope of each curve in the range of 0.00 < *ε* < 0.02, and listed in Table 1, where it can be seen that the [001] orientation is the softest and [111] is the stiffest. This agrees with results of bulk BCC iron [40]. The *E* of the sample under compression is larger than that under tension except for [001] orientation, in consistency with that observed in Cu single crystal [56], which should be ascribed to the higher friction under compression [56]. The rest of curves at the elastic stage under compression deviate from that under tension obviously, which should be ascribed to the asymmetrical tensile and compressive nature of the interatomic potential [57]. After the first peak, it is unclear whether deformation should be attributed to dislocation slip or phase transformation; therefore, this region cannot be seen as inelastic or plastic T/C asymmetry, which differs with others’ works [57–59]. In the flowing section, the deformation mechanisms of the sample subjected to loadings along different directions will be discussed in details.

**Table 1:** *E* of samples under tension and compression along different orientations

	*E*_[001]_ (GPa)	*E*_[110]_ (GPa)	*E*_[111]_ (GPa)
Tension	125.68	150.09	160.99
Compression	124.97	176.23	199.97

### Failure Behavior

**Fig. 3:**
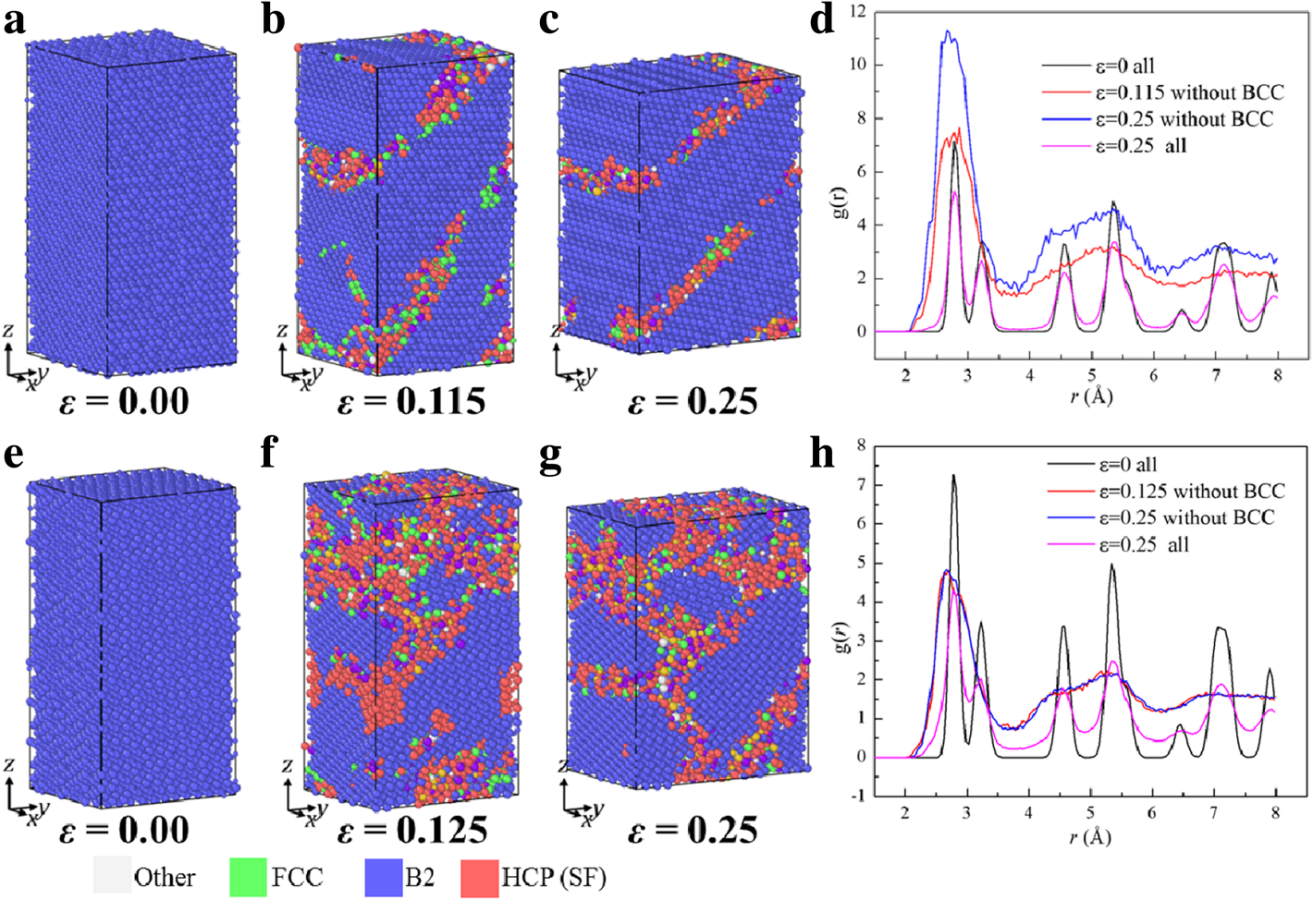
Atomic configurations and RDFs of samples under compression. **a**–**d** Along [110] and **e**–**h** along [111]

Figure 3 shows the atomic structures and the radial distribution functions (RDFs) of the samples subjected to compression along [110] and [111], whose *σ*-*ε* curves can be seen in Fig. 2 labeled as [110] C and [111] C. Figure 3a, d shows the initial samples [110] and [111] after relaxation at 300 K, where it can be seen that the atoms are in the B2 structure. When *ε* is increased to 0.115 or 0.125 for [110] C and [111] C, respectively, the region with mixed structures appears, as shown in Fig. 3b, f. The structure in the mixed area is defined as a mixed phase. The nucleation of mixed phase corresponds to the rapid drops stage of curves [110] C and [111] C in Fig. 2b, c, which is marked with arrows. Therefore, the transformation of local structure from B2 to a mixed phase causes the rapid drop of stress. In the flowing stage, the volume fraction variation of the mixed phase is the main mechanism to accommodate further deformation, the local structures in the samples under compression along [110] and [111] at *ε* = 0.25 are shown in Fig. 3c, g, respectively. To specify the structure in the mixed region, the radial distribution function (*RDFs*), *g*(*r*), of the samples under compression along [110] and [111] at different strains are shown in Fig. 3d, h. The peaks of the *g*(*r*) of the samples at *ε* = 0 and *ε* = 0.25 are sharp, indicating that they still maintain crystalline feature. While the peaks of the *g*(*r*) for the mixed regions, i.e., the sample without B2 regions, are blunt except the first ones, indicating that the mixed region is in an amorphous state. The dislocation extraction algorithm (DXA) [60] are also used to detect whether there is any dislocation nucleation, and find no obvious dislocations throughout the deformation process. Therefore, the amorphization of the B2 phase is conducted to be the main failure mode, resulting in the rapid drop, marked with arrows in Fig. 2b, c.

**Fig. 4:**
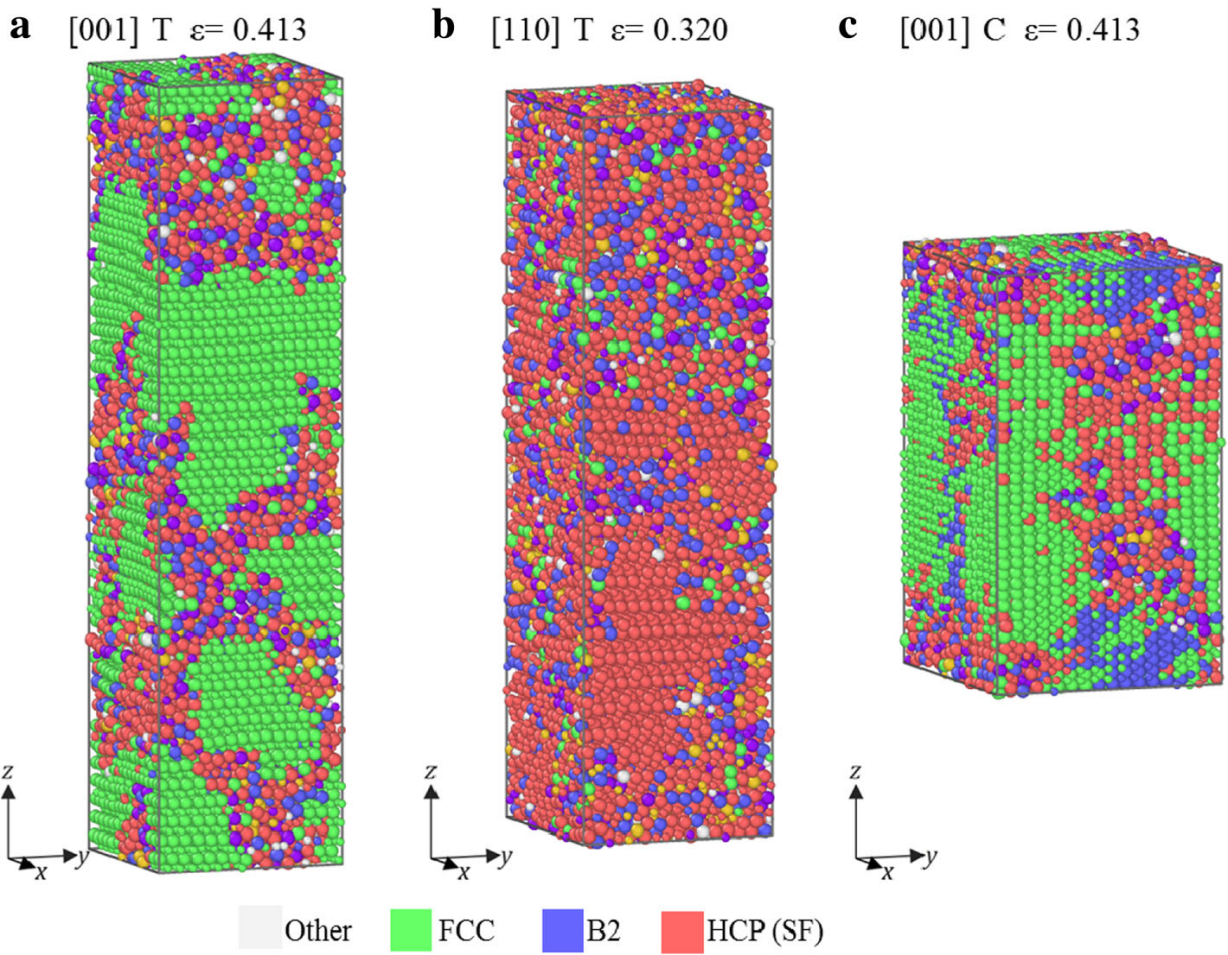
Atomic configurations in samples at failure strain. **a** Under tension along [001], **b** under tension along [110], and **c** under compression along [111]

Figure 4 shows the atomic configurations after the rapid drop strain (failure strain) of the samples under tension along [001], [110] and compression along [001], whose *σ*-*ε* curves belong to group II. It can be seen in Fig. 4 that mixed regions form, similar to that in group I, indicating that amorphization is also the main failure mode (Fig. 4). However, these mixed regions are surrounded by green and red atoms (FCC and HCP structure), which is different from B2 structure in Fig. 3. This difference indicates that the mixed phase is transformed from B2 structure under compression in Fig. 3, but from FCC for sample [001] under tension and compression, and from HCP for sample [110] under tension.

### Phase Transformations

**Fig. 5:**
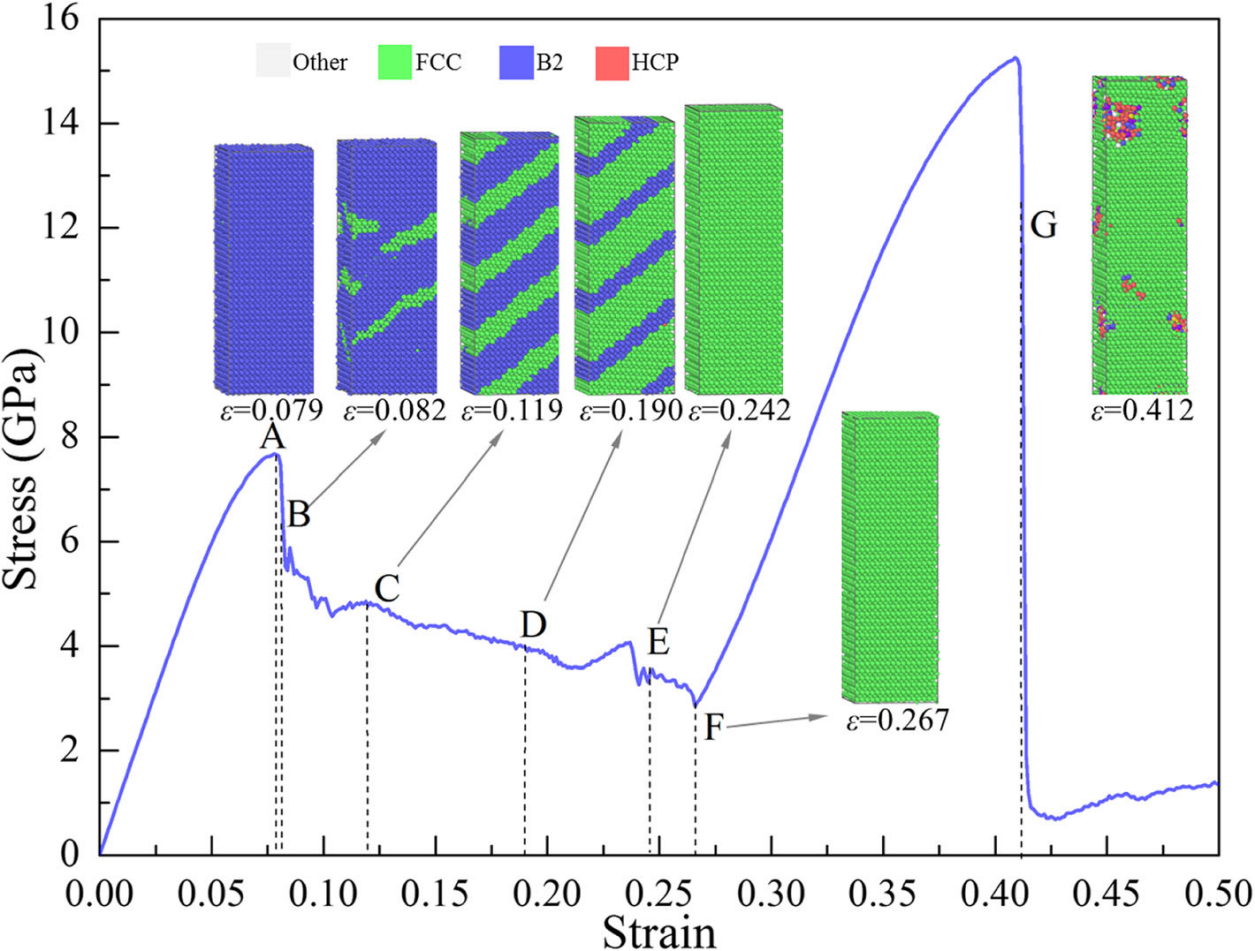
*σ-ε* curve of the sample under tension along [001], colored with local structure, with blue, green, and red representing B2, FCC, and amorphous phases, respectively

**Fig. 6:**
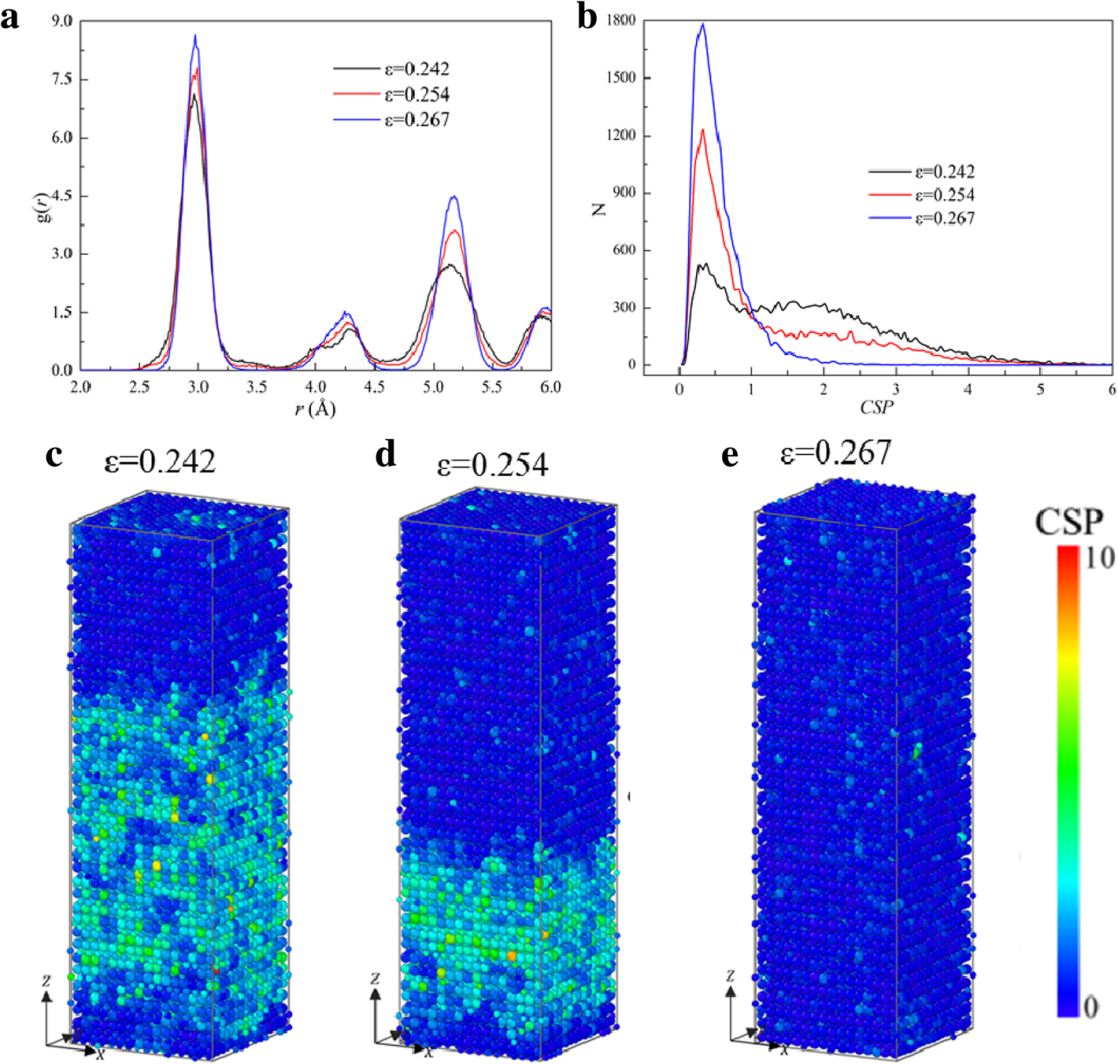
**a** *RDF*, **b** *N*-*CSP* plots of sample under tension along [001]. **c**–**e** Distribution of *CSP* in sample at different strains

Figure 5 shows the *σ*-*ε* curve of the sample [001] under tension, where the microstructures at typical points (marked with A, B, ... , G) are also exhibited. The atoms at point A (*ε* = 0.079) are in B2 structure, indicating that before *ε* = 0.079 the deformation in the sample is elastic. However, local transformation from B2 to FCC takes place, as shown in the inset of *ε* = 0.082 in Fig. 5, resulting in the first rapid drop (A→B) to *ε* = 0.082, where the release of the elastic energy stored due to the redistribution of the atomic configuration provides the energy for the need of the phase transformation. In the flowing stage of B→F, the elastic energy stored is further released as the phase transformation continues, leading to the reduction of stress. The local structure of the sample becomes FCC completely at *ε* = 0.242 (point E). And the structure keeps changed between Point E and F, but the stress keeps falling with the increase of strain. To reveal the change of microstructure, the distribution of *g*(*r*) and the variation of the number of atoms (*N*) against *CSP* (*N*-*CSP*) of the sample at *ε* = 0.242, 0.254, and 0.267 (between points E and F) are calculated and shown in Fig. 6a, b, respectively, where the height of each peak increase with the increase of strain, indicating that the system becomes more compact.

To characterize whether an atom is a part of a perfect lattice or a local defect, e.g., a dislocation or stacking fault, or a surface, the *CSP* [53] of the atoms with the number of nearest neighbors of FCC structure (*N* = 12) are calculated, as shown in Fig. 6c–e. And larger CSP means a larger deviation from the perfect lattice [17]. It can be seen in Fig. 6b that the number of atoms with CSP < 1 increase with the increase of strain, which can be also seen more intuitively from the distribution of CSPs of atoms in Fig. 6c–e. It differs from the common results that usually CSP would not decrease with the increase of strain. Therefore, the main deformation behaviors in this stage should also be attributed to the phase transformation from imperfect FCC to perfect FCC. In the flowing stage, the sample with FCC structures is stretched, and stress will increase until reaching the second yield point. Then the curve drops sharply, corresponding to local amorphization rather than the nucleation of dislocation or slip.

**Fig. 7:**
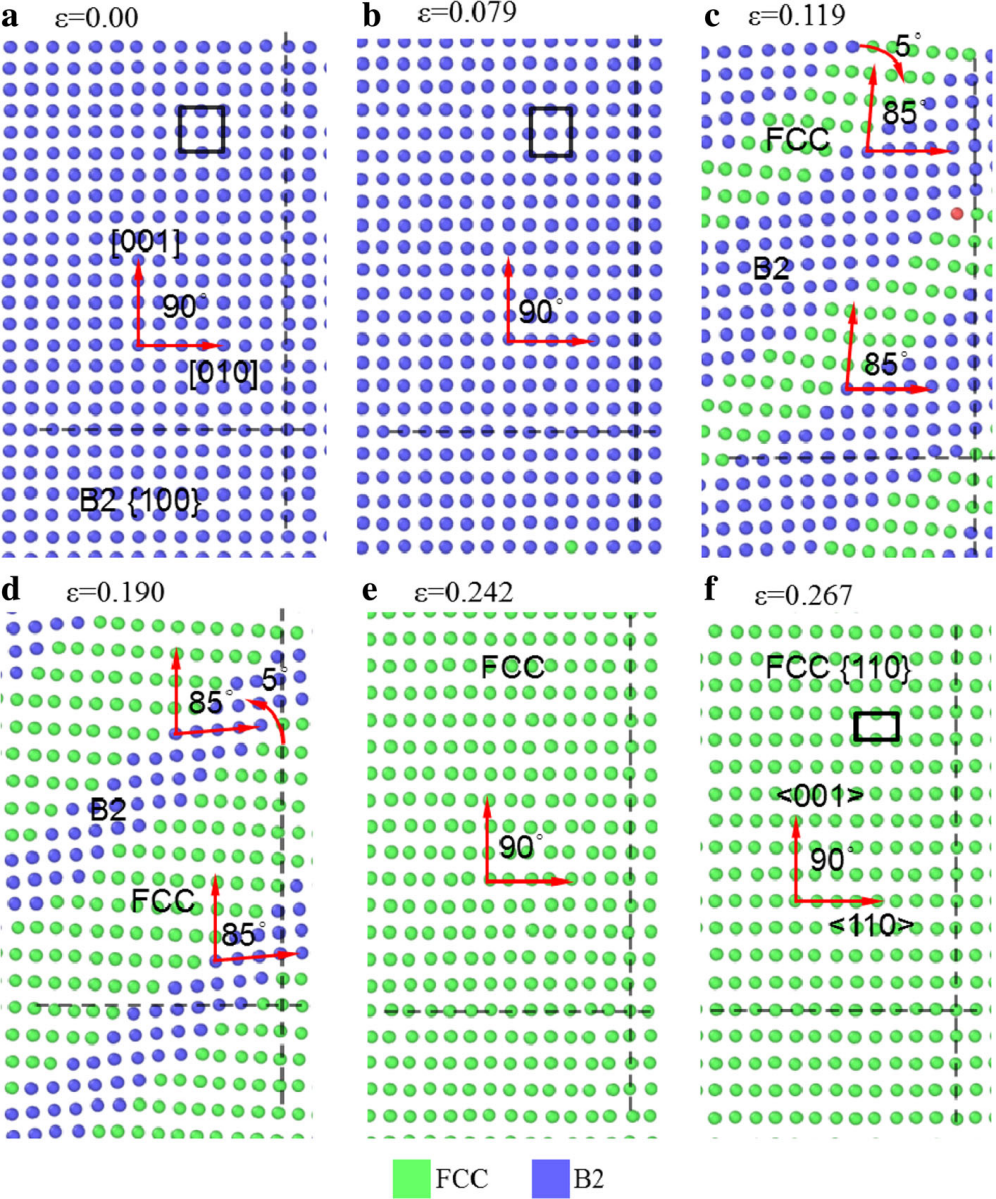
*yoz* slices of [001] sample under tension at different strains, colored with a local lattice structure, with blue, green, and red representing B2, FCC, and amorphous phases, respectively

To illustrate the phase transformation of the material during deformation, Fig. 7 shows some *yoz* slices of the sample [001] under tension at different strains. The horizontal and vertical dashed lines are used as references to identify whether the atomic structures rotate and deviate. With the increase of *ε* from 0.0 to 0.079, the atoms lie on lines parallel to the horizontal and vertical axes, indicating that they are of B2 local structure. However, the atomic array in Fig. 7b changes to that in Fig. 7c as *ε* from 0.079 to 0.119 when some B2 structures transform to the FCC structure. The angle between the arrays changes from 90° in Fig. 7b for B2 structure to ~ 85° in Fig. 7c for FCC structure with the lattice orientation deviating from the vertical axis by clockwise 5°, but the atomic array of B2 structure does not change obviously and has no obvious rotation. During 0.119 < *ε <* 0.190, the FCC area increases, and the array of green atoms gradually rotate counterclockwise. At *ε* = 0.242, all the B2 structure transforms to FCC structures, as shown in Fig. 7e, where the three lattice orientations become nearly parallel with the three axes respectively, but there is still marked deviation, indicating the FCC structure is not perfect, which is in consistency with that shown in Fig. 6c. In the flowing stage, the atomic array tends regularly, as shown in Fig. 7f at *ε* = 0.267, which is recognized as a {110} plane of FCC structure, with its crystal orientation in horizontal and vertical directions changed from [010] and [001] of B2 structure to <110> and <001> of FCC structure.

**Fig. 8:**
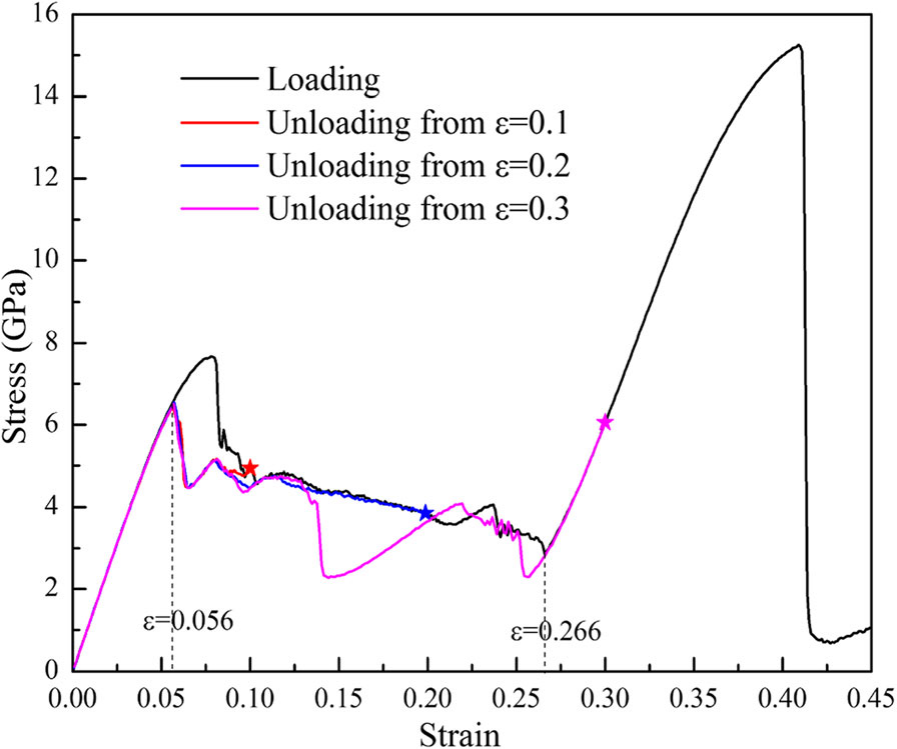
Loading and unloading *σ*-*ε* curves tension along [001] from different strains

The simulation for the response of the sample (100) during unloading from different maximum tensile strains (*ε*_max_ = 0.1, 0.2, and 0.3, respectively) are performed, and the *σ*-*ε* curves are shown in Fig. 8. It can be seen that the unloading *σ*-*ε* curves between *ε* = 0.266 and *ε* = 0.056 do not overlap the loading curve, but they can meet with the elastic *σ*-*ε* curve at *ε* = 0.056 and then return to the origin along the elastic *σ*-*ε* curve, exhibiting the superelastic characteristic. The loading and unloading paths form hysteresis loops, which should be ascribed to the different paths of forwarding and inverse phase transformations.

**Fig. 9:**
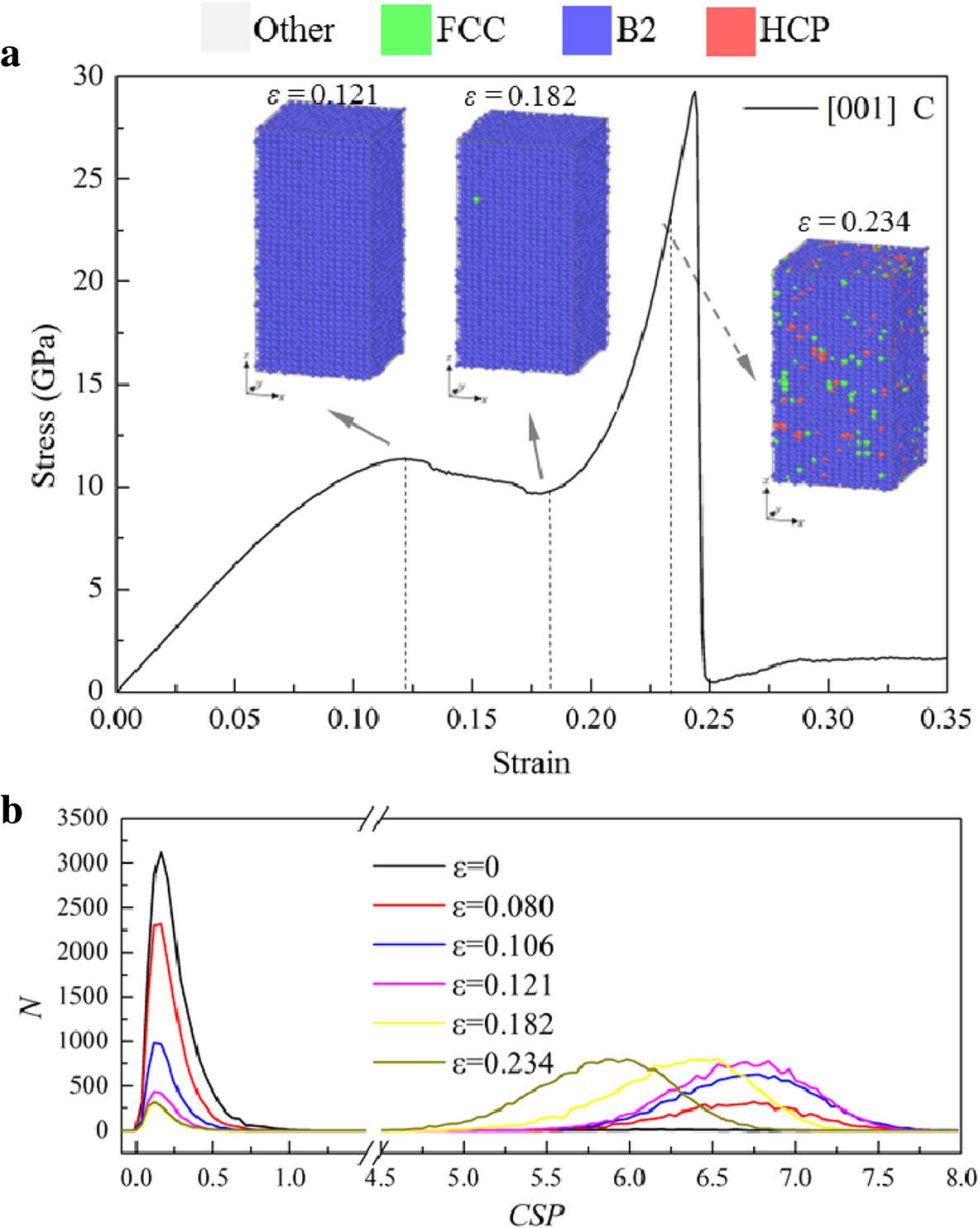
Responses of the sample [001] under compression. **a** *σ-ε* curve and typical atomic configurations, with atoms colored with local structures identified by PTM. **b** *N*-*CSP* plots

**Fig. 10:**
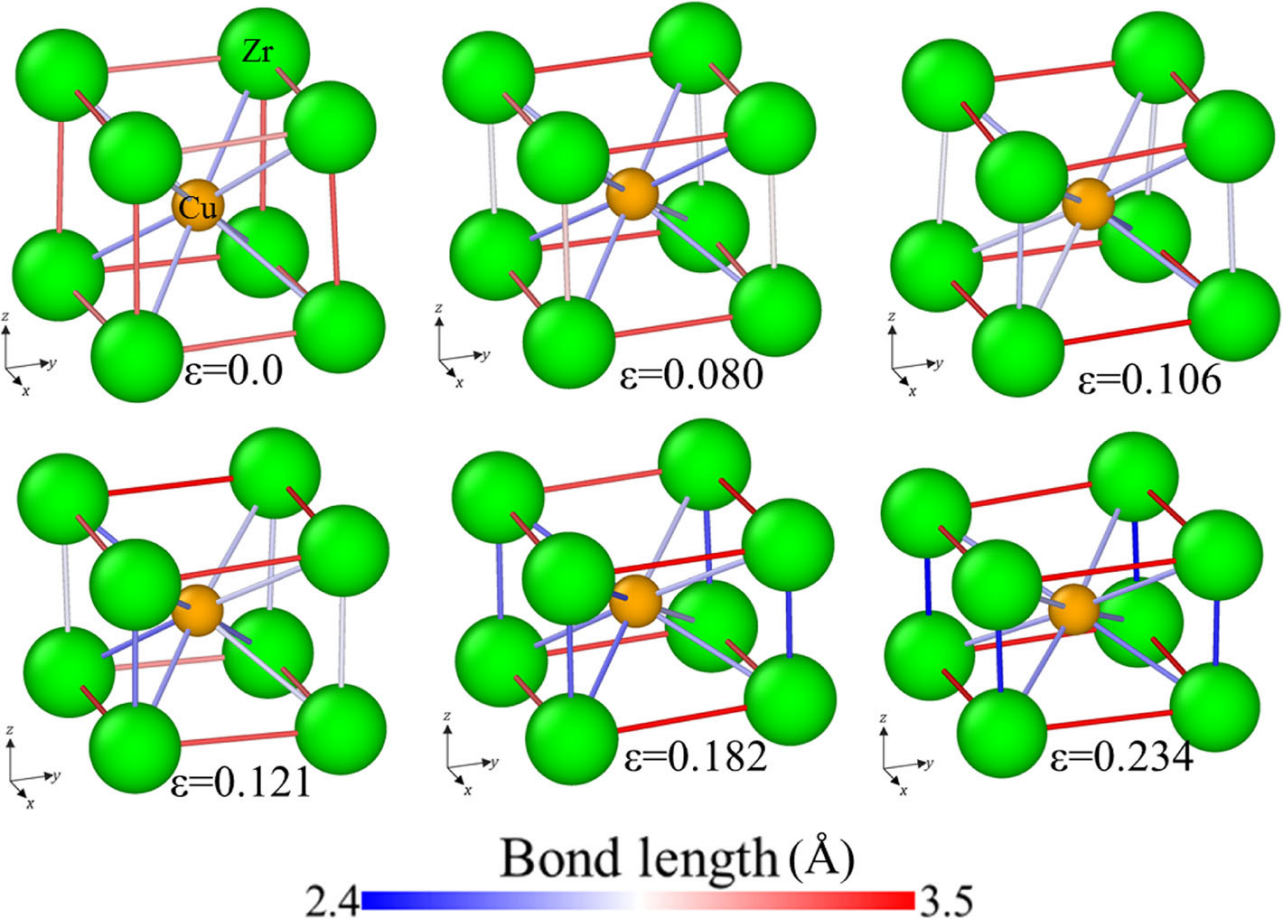
Evolution of bond length for the sample [001] under compression, with bonds colored with its length

The *σ*-*ε* curve of the sample [001] under compression has a similar trend, as shown in Fig. 9a, which can be divided into four stages: (1) *σ* increases linearly with the increase of strain; (2) *σ* drops to a plateau; (3) *σ* increases linearly with a larger slope to the second peak as the increase of strain; (4) *σ* drops sharply to the second plateau. By analyzing the local structures with PTM, one can find that most atoms are identified as B2 structure before reaching the second peak, except some scattered atoms that are identified as other local structure, as shown in Fig. 9a. However, according to the previous understanding, a sudden change in the *σ*-*ε* curves usually corresponds to the change of microstructure. To further confirm it, the *N*-*CSP* plots are calculated and shown in Fig. 9b, in which the CSP for each atom at different strains is calculated with the nearest neighbors of the B2 structure (*N* = 8). When *ε* = 0, the *CSP* of atoms is larger than 0, but less than 1, due to the effect of temperature, implying that the atoms are in perfect B2 structure. With the increase of *ε*, the atoms can be divided into two groups by their CSPs: CSP < 1 and 5 < CSP < 7.5. At 0.080 < *ε <* 0.121, the CSPs at the second peaks are the same, but the number of atoms in this *CSP* range increases and tends stable, indicating the formation of new phases or defects (such as stacking fault). The CSP at the second peak decreases with the further increase of *ε*, i.e., the second wave moves leftwards. Figure 10 shows the evolution of bond length in a unit cell at different strains. At *ε* = 0, eight Cu atoms at the vertexes and one Zr atom at the body-center make up the B2 structure. The relationship between the lattice parameters is *a* = *b* = *c*. With the increase of *ε*, the length of the bond in *xoy* plane increases, but that in *xoz* plane decreases. By calculating the strain in the other two directions during loading, one can find that the strains in the other two directions are identical before the second peaks. Therefore, the lengths of the bonds along *x-* and *y*-directions should be identical, and larger than that along the *z*-direction. The relationship between lattice parameters becomes *a* = *b* > *c*. These arrays of atoms can be recognized as BCT structure. In conclusion, the transformation from a B2 structure to BCT structure is the main deformation mechanism for sample [001] under compression.

**Fig. 11:**
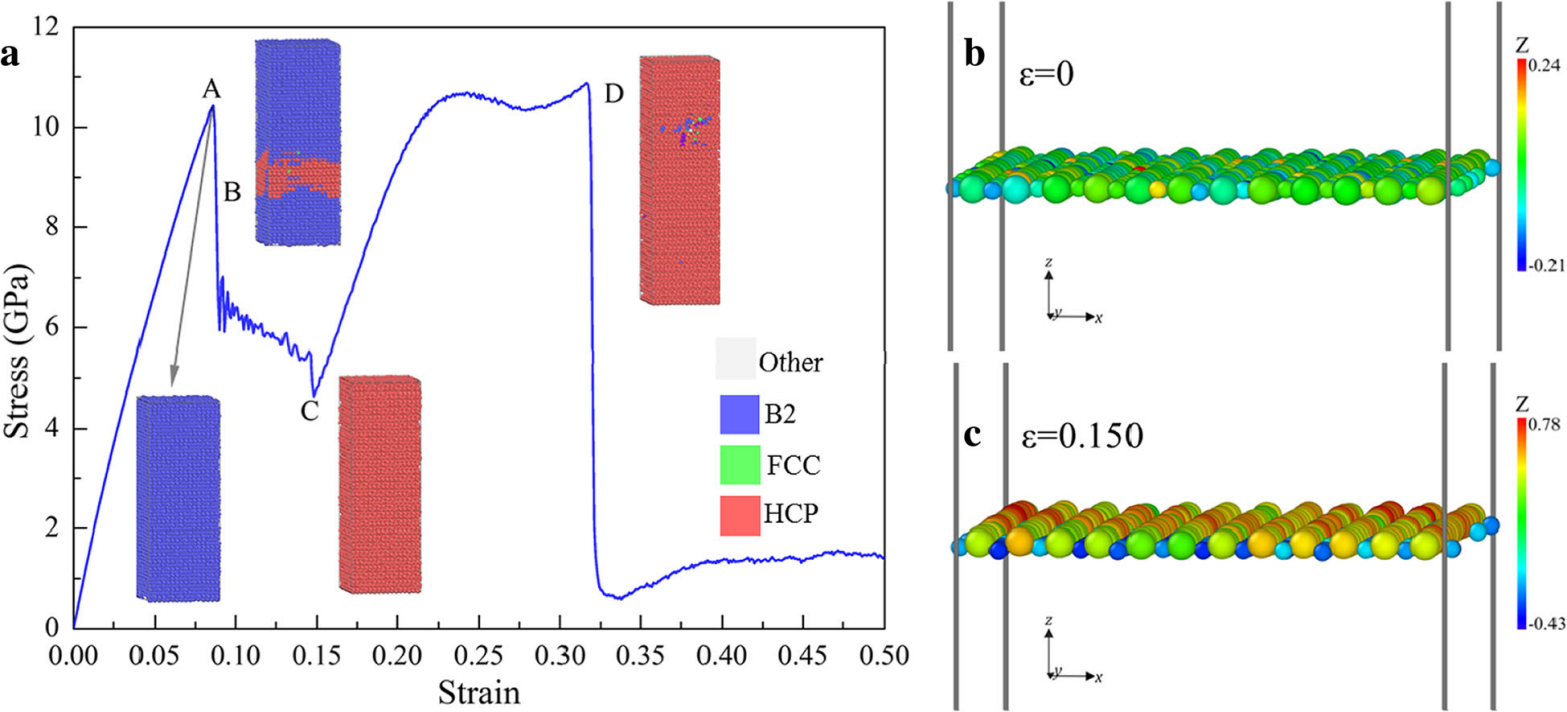
**a** Deformation behavior of tension along [110], colored with PTM. **b**, **c** Atomic slices on *xoy* plane at ε = 0 and 0.150, respectively

Figure 11a shows the response of the sample [110] under tension. The first peak (point A) corresponds to the yield limit of the B2 structure, then some local B2 structure transforms to the HCP structure, resulting in the sharp drop. With the increase of *ε*, the stress decreases until point C when the sample is transformed totally to HCP. Figure 11b, c is the *xoy* one-layer slices at *ε* = 0 and 0.150, respectively, where one can see that at the initial stage the Cu and Zr atoms are in an identical atomic layer (Fig. 11b). However, Cu and Zr atoms are separated into adjacent layers at *ε* = 0.150 (Fig. 11c), which results in the phase transformation from B2 to HCP. Then with the increase of *ε*, *σ* increases with a smaller slope than that in the initial linear stage until point D, followed by a sharp fall to a low stress level, corresponding to failure due to local amorphization.

## Conclusions

In this work, the responses and phase transformation of CuZr samples subjected to uniaxial tension and compression along [001], [110], [111] orientations are studied respectively using molecular dynamics simulations, making use of the latest interatomic potential, from which the following main conclusions can be drawn:

1. The mechanical responses of the CuZr samples under tension and under compression exhibit obvious asymmetry, and their main failure mechanism should be local amorphization.
2. There are three kinds of phase transformations: B2→FCC, B2→BCT, and B2→HCP in tension and compression along [001], and tension along [110].
3. B2→FCC, B2→BCT, and B2→HCP phase transformations are found to be realized by unique mechanisms respectively which are lattice rotation (~ 5°), uniform deformation and separation from Cu and Zr atomic layer for each.
4. Phase transformation region can be recovered after unloading before local amorphization, showing the superelasticity.

The results are important for the exploration of the mechanical properties and deformation mechanisms of nanocrystalline CuZr, and for the applications of nanocrystalline CuZr particles as an enhancement agent to improve the toughness of metallic glass.

## Data Availability

The datasets used or analyzed during the current study are available from the corresponding author on reasonable request.

## Abbreviations

BCC: Body-centered cubic
BCT: Body-centered-tetragonal
BMG: Bulk metallic glasses
BMGC: Bulk metallic glass composites
CG: Conjugate gradient
CNA: Common neighbor analysis
CSP: Centro-symmetry parameter
Cu: Copper
CuZr: Copper-zirconium
DXA: Dislocation extraction algorithm
EAM: Embedded-atom method
FCC: Face-centered cubic
HCP: Hexagonal close-packed
MD: Molecular dynamics
NPT: Constant number of particles, pressure, and temperature
PTM: Polyhedral template matching
RDF: Radial distribution function
T/C: Tension and compression
VN: Vanadium nitride
Zr: Zirconium
*ε*: Strain
*σ*: Stress
